# Experimental and Modeling Studies of Local and Nanoscale *para*-Cresol Behavior: A Comparison of Classical Force Fields

**DOI:** 10.1021/acs.jpca.2c08022

**Published:** 2023-04-11

**Authors:** Katie S. C. Morton, Alin M. Elena, Jeff Armstrong, Alexander J. O’Malley

**Affiliations:** † Institute for Sustainability, University of Bath, Bath BA2 7AY, United Kingdom; ‡ UK Catalysis Hub, Research Complex at Harwell, Science and Technology Facilities Council, Rutherford Appleton Laboratory, Oxford OX11 0FA, United Kingdom; ∥ Daresbury Laboratory, STFC, Daresbury WA4 4AD, United Kingdom; § ISIS Pulsed Neutron and Muon Facility, Science and Technology Facilities Council, Rutherford Appleton Laboratory, Didcot OX11 0QX, United Kingdom

## Abstract

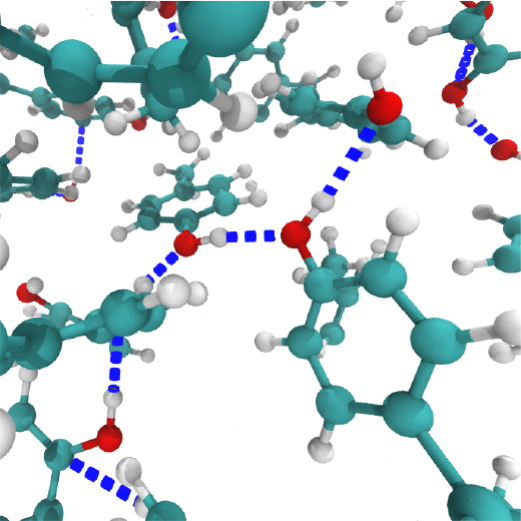

The dynamics of bulk liquid *para*-cresol from 340–390 K was probed using a tandem quasielastic neutron scattering (QENS) and molecular dynamics (MD) approach, due to its relevance as a simple model component of lignin pyrolysis oil. QENS experiments observed both translational jump diffusion and isotropic rotation, with diffusion coefficients ranging from 10.1 to 28.6 × 10^–10^ m^2^s^–1^ and rotational rates from 5.7 to 9.2 × 10^10^ s^–1^. The associated activation energies were 22.7 ± 0.6 and 10.1 ± 1.2 kJmol^–1^ for the two different dynamics. MD simulations applying two different force field models (OPLS3 and OPLS2005) gave values close to the experimental diffusion coefficients and rotational rates obtained upon calculating the incoherent dynamic structure factor from the simulations over the same time scale probed by the QENS spectrometer. The simulations gave resulting jump diffusion coefficients that were slower by factors of 2.0 and 3.8 and rates of rotation that were slower by factors of 1.2 and 1.6. Comparing the two force field sets, the OPLS3 model showed slower rates of dynamics likely due to a higher molecular polarity, leading to greater quantities and strengths of hydrogen bonding.

## Introduction

Cresols (methylphenols) are significant intermediates in the catalytic transformation of fuels and value-added chemicals from biomass feed stocks. In numerous industrial processes, cresols are present as both desirable and undesirable components. Their existence in soils and water supplies poses a major threat to the environment and human health due to their toxic, mutagenic, and carcinogenic properties.^1−3^ They are abundant in the lignin component of biomass, constituting 20.6 wt % of lignin pyrolysis products in a conversion study performed by Rezaei and colleagues, which was further increased through catalytic depolymerization methods.^4^ In a study of the relative production of each isomer type, approximately twice the amount of the *meta*- and *para*-isomers compared to the *ortho*-isomer was obtained from several different lignin sources.^5^*p*-Cresol also forms the largest component of the problematic tar that forms during biomass gasification,^6^ but extraction technologies may aid the purification or removal of unwanted cresol contaminants.^7^

In this paper the local and long-range diffusion mechanisms of *para*-cresol (4-methylphenol) have been studied to gain insight into important behavior associated with the extraction, separation, and catalytic conversion of a significant species (and an important model component) in the complex mixtures associated with biomass pyrolysis. Studying cresol mobility, and the development of accurate computational models describing their intermolecular interactions, precedes any of the physico-chemical studies associated with the design and optimization of homogeneous and heterogeneous catalytic processes for their conversion to more desired chemicals—with mass transport being a common rate-limiting process.^8^*p*-Cresol is a common precursor for producing value-added chemicals including dyes,^9^ polymers^10^ and benzene, toluene and xylene (BTX) petrochemicals.^11^*p*-Cresol derivatives including butylated hydroxyl toluene are employed in the manufacture of phenolic resins,^12^ while others such as *p*-hydroxybenzonitrile are agrochemical precursors^13^ and anisaldehyde in diltiazem or trimethoprim for making pharmaceuticals.^14^ The procurement of distinct *p*-cresol product fractions simplifies its catalytic transformation, but the similarities in the volatilities of the *meta*- and *para*-isomers in particular (boiling points of 202.0 and 202.3 °C respectively) complicate their separation. Many reported cresol extraction procedures rely upon differences in adsorption affinity, geometry, and diffusion properties.^15,16^ Successful isomeric separations have been performed with shape-selective zeolite catalysts^17,18^ and adsorbent mixed-matrix membranes.^19^

In terms of probing fundamental molecular behavior, the internal methyl rotations of cresols have been studied using density functional theory (DFT) and microwave spectroscopy.^20,21^ Previous molecular dynamics (MD) simulations have modeled their densities and heats of vaporization in accordance with experimental studies.^22,23^ To the best of our knowledge, the mobility of a pure liquid cresol has not been studied. The Taylor dispersion method was employed to measure the diffusion coefficients of all three cresol isomers but in an aqueous medium, recording values from 15.8–16.1 × 10^–9^ m^2^s^–1^ at 323 K, with the *p*-isomer diffusing the fastest through water due to its lower steric hindrance.^24^

Quasielastic neutron scattering (QENS) studies have shown the effect of methyl and hydroxyl substituents on the diffusion of aromatics—with the diffusion coefficients of benzene, toluene, and phenol being 3.48, 4.50 × 10^–9^ m^2^s^–1^ at 333 K and 0.23 × 10^–9^ m^2^s^–1^ at 318 K, respectively.^25^ All of the aromatic species displayed diffusion via a jump diffusion mechanism, whereby the molecules oscillated in a fixed position before jumping to a new equilibrium position. The relative effect of the addition of a hydroxy versus a methyl functional group to benzene on its diffusion highlights the significance of hydrogen bonding in reducing the diffusivity. In the same study, inelastic neutron scattering was also applied to investigate the rotational frequencies of the aforementioned molecules. Each liquid exhibited a frequency associated with aromatic ring rotation around an axis within the molecular plane that increased with temperature, but its rotation around a perpendicular axis was temperature independent. Liquid phenol displayed additional vibrations associated with the hydroxy protons when involved in hydrogen bonding with adjacent molecules and when oscillating after the breaking of a hydrogen bond. Toluene also has an additional frequency associated with its methyl group rotation.

Studying the structure of aromatic liquids displaying some degree of crystallinity at lower temperatures (termed “quasi-crystallinity”) may aid the understanding of the local structures that may hinder the rotation and translation of aromatic *p*-cresol molecules. The small aromatic molecule benzene most commonly conformed to a right-angled arrangement at 298 K determined by X-ray diffraction.^26^ A neutron diffraction study confirmed a similar structure with strong bonding between the aromatic ring of one benzene and either one (“T-shaped”) or more likely two (“Y-shaped”) of the ring hydrogen atoms of an adjacent perpendicular molecule.^27,28^ However, due to the presence of the hydroxyl group in *p*-cresol, hydroxyl-π interactions may also occur, as Takahasi et al. found that more acidic protons interact with the π-system of aromatic rings more strongly than aromatic protons and tend toward the 180° of hydrogen bonding.^29^ Other local structures of small aromatic molecules included offset parallel stacking formations through π-π interactions and trimers formed with molecules at 60° to one another.^27^ The additional methyl functionality of cresols may contribute to a more disordered liquid structure, as an increase in disorder was detected in liquid toluene compared to benzene.^27^ However, the ability of cresols to form hydrogen bonds may increase the degree of order.

A combined QENS and MD simulation approach is uniquely equipped for studying qualitative and quantitative aspects in both liquid state physics and adsorbate dynamics in heterogeneous catalysis.^30,31^ Neutron sensitivity to ^1^H enables the direct measurement of cresol motions, across time scales of picoseconds to nanoseconds, relating to translational and rotational energies. This time scale is comparable to MD studies, unlike other macroscopic methods used to measure diffusion. In catalysis, the study of both long-range diffusion and local dynamics is vital for studying mass transport and confinement effects, which will be affected by intermolecular interactions at high loadings. Notably, QENS observables concerning liquid cresol motion can provide an important aid in the parametrization of classical force fields on which the MD simulations are based, allowing us to improve on existing force fields that may encounter issues when simulating bulk dynamics.^32^

In this study, MD simulations based on two different OPLS force field models (OPLS3^33^ and OPLS2005^34^) were carried out in tandem with QENS experiments to compare the effects of the classical potentials employed on the local dynamics and nanoscale diffusion of *p*-cresol. QENS observables were then generated from MD simulations through calculation of the simulated incoherent dynamic structure factor (*S*_*inc*_(*Q*, ω)) for a direct comparison to experiment, to test the accuracy of the different classical force field models over the time scale of the QENS instrument. We find both notable consistencies and discrepancies in the cresol behaviors and rates observed between experiment and modeling.

## Methods

### Quasielastic Neutron Scattering Experiments

Commercial *p*-cresol samples were obtained from Sigma-Aldrich (CAS number: 106-44-5). Under a helium atmosphere, the *p*-cresol in liquid form was loaded into an indium wire sealed, annular, thin-walled, aluminum can with a 0.25 mm annulus to give around 10% scattering (to minimize the contributions of multiple scattering to the total signal). QENS experiments were performed on the time-of-flight, backscattering instrument IRIS, at the ISIS Pulsed Neutron and Muon source, Rutherford Appleton Laboratory, Oxfordshire. A graphite 002 analyzer crystal gave an energy resolution of 17.5 μeV. Energy transfers were measured within ±0.53 meV across a *Q* range of 0.42–1.85 Å^–1^. QENS spectra were obtained at 340, 370, and 390 K; this temperature range was selected by considering the catalytic temperatures typically applied in cresol conversion and to avoid any cresol decomposition or melting of the indium seal. Differences in detector efficiency were accounted for by calibrating all of the samples against a vanadium standard measurement. The same vanadium measurements were also used as the resolution function for the instrument. MANTID^35^ and DAVE^36^ neutron scattering analysis software packages were used to fit the QENS data. The detectors were averaged into 11 groups from *Q* = 0.48 to 1.53 Å^–1^. A delta function was included in the fitting process to account for scattering from the aluminum can. Time limitations did not allow for adequate measurements of the empty can.

### Molecular Dynamics Simulations

#### Building and Running the Simulated Systems

Initially a single *p*-cresol molecule was geometry optimized by DFT via the program Gaussian,^37^ employing the hybrid functional B3LYP which uses the gradient corrected Becke88 exchange functional^38^ and the correlation function of Lee, Yang, and Parr.^39^ The QZVP basis set was applied.^40^ Next, a bulk liquid configuration was generated using Packmol software^41^ which placed 384 geometry optimized *p*-cresol molecules (>2 Å apart) into a cube of dimensions 40 × 40 × 40 Å^3^.

The cresol atomic charges and the intramolecular and intermolecular potentials were taken from OPLS 2005^34^ and OPLS3^33^ respectively, as shown in Table S1 in the Supporting Information. Initial field files for cresol systems were generated using DL_FIELD 4.6.^42^ The potentials were derived from Monte Carlo simulations optimized to match experimental data such as the heat of vaporization and density.^43−45^ The two models differ in their atomic charges, in particular the hydroxyl group charges, which are 2–3% larger in the OPLS3 model. The charges related to each atom are displayed in Figure S1. The partial atomic charges in the OPLS2005 model were assigned by the distribution of any formal ionic charges over one or more atoms, combined with contributions from bond charge increment parameters associated with chemical bonds to ensure the net charge of the molecule maintained neutrality. A CM1A-BCC based charge model is employed in the OPLS3 force field set based on the Cramer-Truhlar CM1A charges with fitted bond charge corrections (BCC). The intramolecular flexibility was modeled by applying harmonic bonds and angles and triple cosine dihedral potentials. The intermolecular energies were described using a combination of Coloumbic contributions and short-range repulsion and dispersion forces calculated by nonbonding Lennard-Jones potentials.

**Figure 1:**
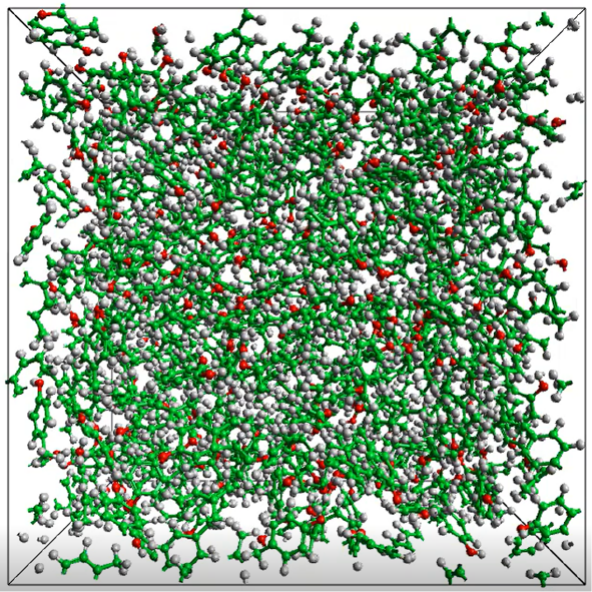
A system of 384 molecules of *p*-cresol equilibrated at 340 K.

All of the MD simulations were run at 340, 370, and 390 K using the DL_POLY 4 code,^46^ parallelized across eight MPI processes. Periodic boundary conditions were used with a cutoff of 10 Å, and the Coulombic relations were treated via the smooth particle mesh Ewald method.^47^ A time step of 0.5 fs was used. Initially, the system was energy minimized and equilibrated during a 1 ns run in the NVT ensemble, followed by a 2 ns run in the NPT ensemble. From this, the average box dimensions were obtained and applied to new cubic systems with recalculated densities, available in Table S2, alongside the measured experimental densities. The new systems were equilibrated for 2 ns of an NVT ensemble. A system equilibrated at 340 K is shown in Figure 1. The production simulations were run for 5 ns in the NVE ensemble following a short equilibration period of 200 ps to the new ensemble. All systems requiring a thermostat or barostat used the Nosé–Hoover variation, with coupling constants of 0.1 and 0.05 ps, respectively. The atom positions were recorded every picosecond.

#### Analysis of the Molecular Dynamics Simulations

The mean squared displacements (MSDs) were obtained from the atom trajectories weighted by the incoherent scattering of each atom to observe the bulk diffusion of cresols over longer time scales, via the program MDANSE.^48^ The bulk self-diffusion coefficients (*D*_*s*_) were calculated using the Einstein relation (eq 1) from 100 to 4000 ps, where the log(MSD)–log(*t*) relationship was linear:
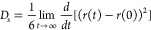
1where *r* is the position of the center of mass of the molecule in 3D space and *t* is time.

The dynamics of *p*-cresol molecules within the MD simulations were directly compared to the experiment by calculating the simulated incoherent dynamic structure factor (*S*_*inc*_(*Q*, ω)) using MDANSE.^48^ MDANSE calculates *S*_*inc*_(*Q*, ω) through calculation of the incoherent part of the total intermediate scattering function (ISF) for each simulation via the following expression:
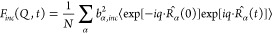
2where *N* is the number of atoms,  are the position operators of the atom nuclei, and *b*_α,*inc*_ is the nuclei incoherent scattering lengths. The angular brackets represent an NVE ensemble averaged over the set of initial times.

The relation between the incoherent ISF and the incoherent dynamical structure factor (DSF) is shown below. The self-part of the ISF undergoes a Fourier transformation in the frequency domain:
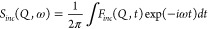
3

The simulated incoherent DSFs were fit by DAVE in the same way as the QENS data. To directly compare this with the QENS experiment, during the calculation of *S*_*inc*_(*Q*, ω), a resolution function matching that of the IRIS instrument was applied as a Gaussian curve with a full-width half-maximum (fwhm) equal to 17.5 μeV. As with the experiment, the data were fit across an energy transfer range of ±0.53 meV and from *Q* = 0.4–1.6 Å^–1^, sampling every 0.2 Å^–1^.

The radial distribution functions (RDFs) between the O and H hydroxyl atoms on different cresol molecules were calculated by using Visual Molecular Dynamics software.^49^ The same program was used to calculate the number of hydrogen bonds at each time step, where a hydrogen bond was recognized as hydroxyl groups between two different cresol molecules having an O–H–O angle of 180° ± 20° and with the oxygen atoms being within 3.5 Å of one another. The O to O cutoff distance was set based on the location of the minima after the first peak observed in the oxygen to oxygen RDF plots. The mean number of hydrogen bonds occurring per time step per molecule across the course of the simulation was then calculated. First the average number of hydrogen bonds per time step for each system was determined and then divided by half the number of molecules present in the simulation, as two molecules are involved in a single hydrogen bond.

## Results and Discussion

### Quasielastic Neutron Scattering

**Figure 2:**
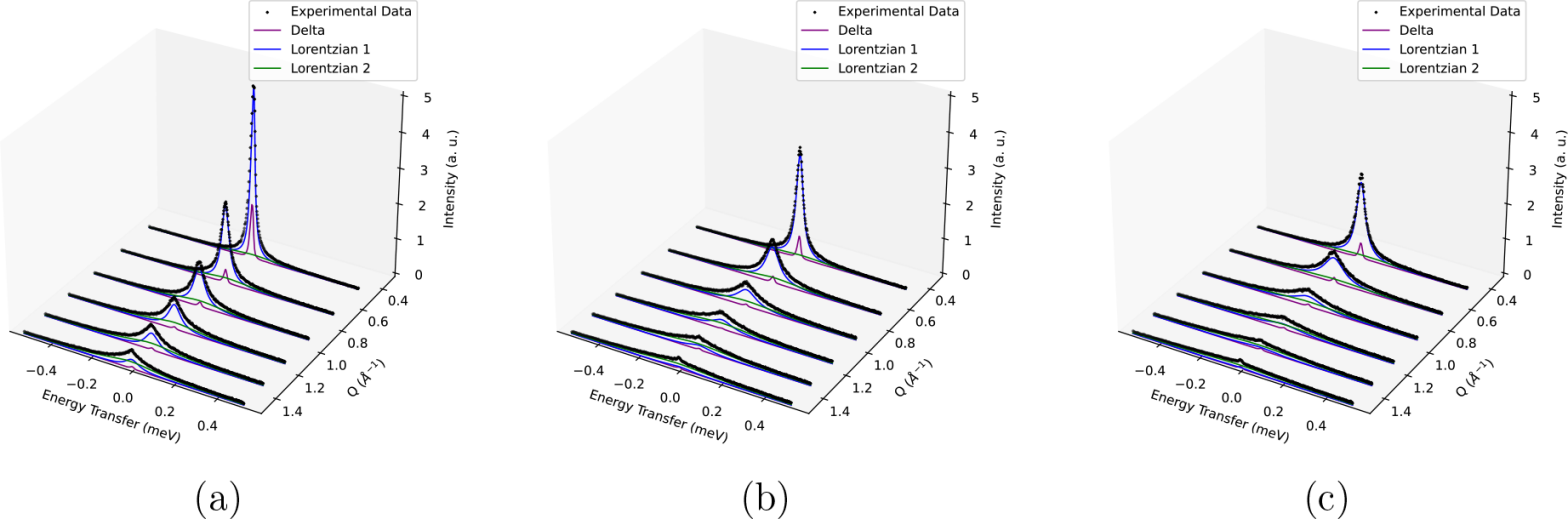
QENS spectra as a function of *Q* for *p*-cresol at (a) 340, (b) 370, and (c) 390 K.

Significant broadening of the QENS spectra is observed at all *Q* values for liquid *p*-cresol from 340 to 390 K in Figure 2. The detectors varying with *Q* were averaged into 11 groups, 6 of which are shown at each temperature for clarity. 2D QENS spectra showing the Lorentzian fittings in greater detail for different values of *Q* at 370 K can be found in Figure S3.

A flat background, delta function, and two Lorentzian functions were convolved with the resolution function, which were required to fit each QENS spectrum adequately. The presence of a background suggests that fast motions such as hydroxyl and methyl rotations were occurring outside the time scale of the instrument and also accounts for the Debye–Waller factor.^50^ The delta function mostly accounts for the elastic scattering from the aluminum can. Two Lorentzians of significantly different widths labeled “Lorentzian 1” and “Lorentzian 2” describe the quasielastic broadening, suggesting two types of motions were dominant on the time scale of the instrument. The contribution from both Lorentzian components increases with temperature, indicating faster/more movement.

**Figure 3:**
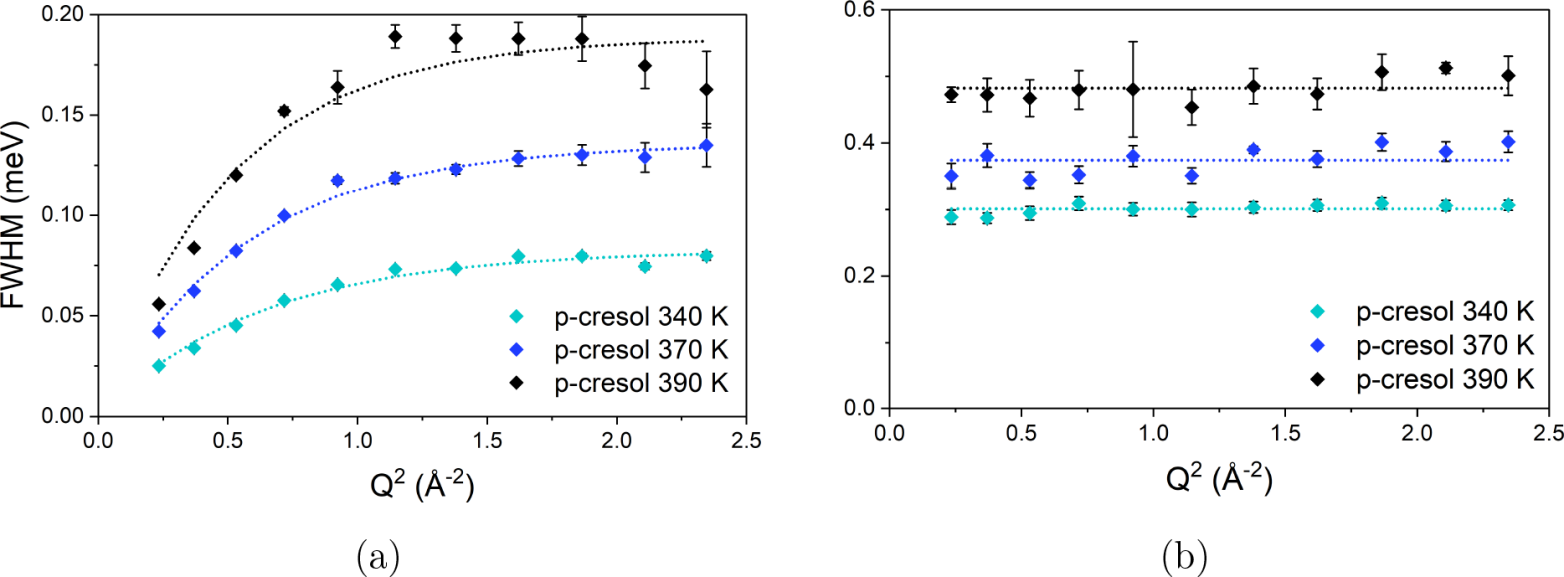
Obtained from QENS spectra of *p*-cresol from 340 to 390 K, the *Q* dependence of the fwhm of (a) Lorentzian 1 fit by models of Hall–Ross jump diffusion and (b) Lorentzian 2 fit by models of isotropic rotation.

For cresol molecules, the dynamics likely incorporate long-range translation and local dynamics, such as confined translation or rotations. The variation of each Lorentzian fwhm with *Q* can be fit with models describing the type and rate of the associated motion. Details of the models and their mathematical forms for fitting the Lorentzian fwhm are discussed in the Supporting Information. The fwhm of each Lorentzian, plotted as a function of *Q*, is shown in Figure 3.

The fwhm of Lorentzian 1 relates to translational diffusion, as it exhibits an initial linear proportionality to *Q*^2^ at low *Q*. A plateau is reached at high *Q*^2^, typical of a jump-diffusion process, which has been observed for other simple aromatic species.^25,51^ The Hall-Ross model of jump diffusion was fit to the data, which is characterized by a residence time where the cresol molecules vibrate around an equilibrium position followed by a jump with a Gaussian distribution of jump lengths. The width of Lorentzian 2 shows no trend with *Q*, indicative of a localized rotational motion. This rotational motion is likely the isotropic rotation of the whole molecule around any axis, which has been observed in QENS experiments on other simple aromatic molecules such as the isotropic rotation of phenol within zeolite beta measured from 2.6 to 11.5 × 10^10^ s^–1^ at 393 K and activation energies for rotation of 7–14 kJmol^–1^.^50^ The jump diffusion and resulting diffusion coefficients for the translational motion are displayed in Table 1 along with the activation energies (*E*_*a*_). The rotational rates and associated activation energies are displayed in Table 2. Also displayed are the values for the same motions obtained from the DSF derived from MD simulations using the same analysis process that was applied to the DSF obtained from the QENS experiment. The MD derived results are discussed in more detail in the following sections.

**Table 1:** Self-Diffusion Coefficients (*D*_*s*_) and the Activation Energies (*E*_*a*_) for the Jump Diffusion of *p*-Cresol, Calculated from QENS Experiments, and the DSF Obtained from MD Simulations Applying OPLS2005 and OPLS3 Models

System	Temperature (K)	*D*_*s*_ × 10^–10^ (m^2^s^–1^)	Residence time (ps)	Jump distance (Å)
QENS Experiment	340	10.11 ± 0.01	15.96 ± 0.35	1.80 ± 0.02
	370	18.40 ± 0.59	9.68 ± 0.55	1.89 ± 0.08
	390	28.58 ± 0.64	6.98 ± 0.48	2.00 ± 0.09
	***E***_***a***_**(kJmol**^**–1**^**)**	22.69 ± 0.55		
MD-DSF derived: OPLS2005	340	3.78	18.43	1.18
	370	11.35	9.27	1.45
	390	17.01	8.07	1.66
	***E***_***a***_**(kJmol**^**–1**^**)**	33.73		
MD-DSF derived: OPLS3	340	1.85	50.17	1.36
	370	6.27	17.32	1.47
	390	9.44	9.08	1.31
	***E***_***a***_**(kJmol**^**–1**^**)**	36.66		

**Table 2:** Rotational Coefficients (*D*_*r*_) and the Activation Energy (*E*_*a*_) for the Isotropic Rotation of *p*-Cresol, Calculated from QENS Experiments, and the DSF Obtained from MD Simulations Applying OPLS2005 and OPLS3 Models

System	Temperature (K)	*D*_*r*_ × 10^10^ (s^–1^)
QENS Experiment	340	5.72 ± 0.35
	370	7.10 ± 0.52
	390	9.16 ± 1.06
	***E***_***a***_**(kJmol**^**–1**^**)**	10.07 ± 1.24
MD-DSF derived: OPLS2005	340	4.62
	370	7.00
	390	7.30
	***E***_***a***_**(kJmol**^**–1**^**)**	10.57
MD-DSF derived: OPLS3	340	3.00
	370	4.74
	390	6.57
	***E***_***a***_**(kJmol**^**–1**^**)**	17.14

With temperature, the jump distance increases, and the residence time decreases, leading to the commensurate increase in *D*_*s*_. The jump distances are slightly less than the radius of one cresol molecule (ca. 2.9 Å), and therefore the jumping must be more incremental than the complete displacement of a cresol molecule out of a surrounding “molecular cage”. Previous diffraction studies on liquid benzene have identified structures from which incremental jumping could occur in liquid cresol systems, including “Y” and “T-shaped” dimers and parallel stacking discussed in detail in the introduction.^27^ Also, due to the hydroxyl group present on cresols, none, one, or two hydrogen bonds can be formed per molecule, which could contribute to a complex hydrogen bonded network, where cooperative dynamics and incremental diffusion/reorientation is likely present.

**Figure 4:**
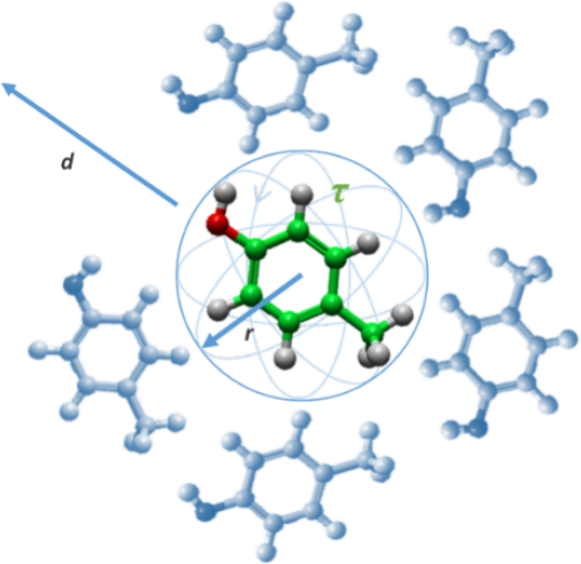
Isotropic rotation of a *p*-cresol molecule around its center of mass with a radius (*r*), during a period of residency (τ), followed by a jump of a certain distance (*d*) when undergoing jump diffusion.

The activation energies were calculated from the Arrhenius plots found in Figure S6. As expected, the activation energy for jump diffusion was greater than that for isotropic rotation. Dervin *et al.* measured faster rates of jump diffusion in liquid benzene from 3.4–5.0 × 10^–9^ m^2^s^–1^, even at lower temperatures (300–350 K) and an associated activation energy of 7.1 kJmol^–1^ was calculated. Therefore, the addition of hydroxyl and methyl functional groups leads to a slower rate of translation and a higher activation energy for jump diffusion, likely due to stronger bonding interactions and/or steric effects.^51^Figure 4 illustrates the possible mechanism of a molecule of *p*-cresol undergoing these simultaneous motions.

### Molecular Dynamics Simulations

To compare with the QENS experiment above, MD simulations based on two different OPLS force field models were carried out to compare the effects of the classical potentials employed on the local dynamics and nanoscale diffusion of *p*-cresol. In the following section, we have investigated the bulk diffusion of liquid *p*-cresol observed over a much longer time scale (4 ns) than was accessible by the QENS spectrometer by plotting the MSDs. Then the simulations were directly compared with the QENS experiment by calculating the simulated incoherent DSF (*S*_*inc*_(*Q*, ω)), equivalent to that obtained within the experiment, to investigate the accuracy of the different classical force field models over the time scale of the QENS instrument.

#### Mean Squared Displacements

**Figure 5:**
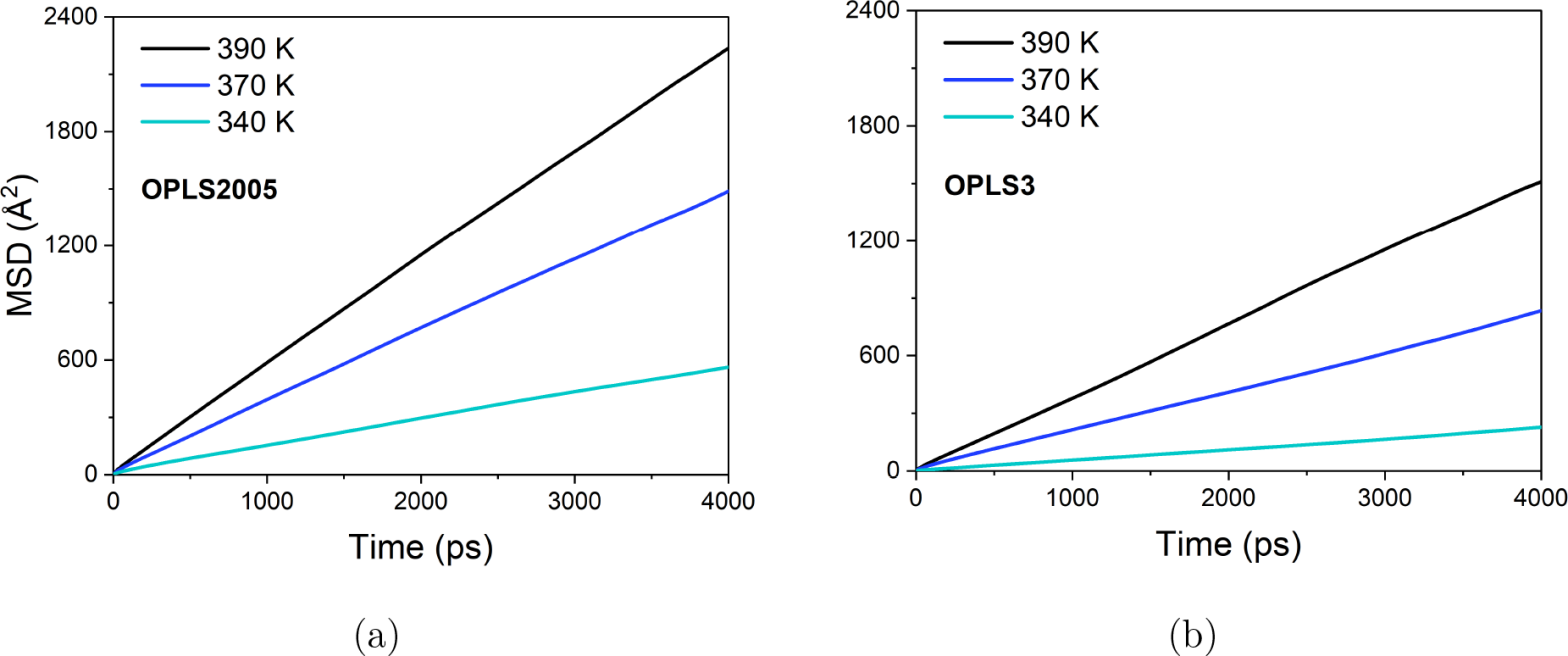
MSD plots of liquid *p*-cresol from 340 to 390 K averaged over 4 ns of MD simulation applying (a) OPLS2005 and (b) OPLS3 potentials.

To observe the bulk translational diffusion of *p*-cresol over longer time scales, the MSD of *p*-cresol from 340 to 390 K for the OPLS2005 and OPLS3 simulated systems over 4 ns were plotted, as shown in Figure 5a,b, respectively. The self-diffusion coefficients (labeled MD-MSD derived) are listed in Table 3 along with their activation energies of diffusion.

**Table 3:** Self-Diffusion Coefficients (*D*_*s*_) of *p*-Cresol Calculated from MSD Plots Obtained from the MD Simulations Applying OPLS2005 and OPLS3 Models from 340 to 390 K and Their Associated Activation Energies (*E*_*a*_)

	MD-MSD derived	
Temperature (K)	OPLS2005 *D*_*s*_ × 10^–10^ (m^2^s^–1^)	OPLS3 *D*_*s*_ × 10^–10^ (m^2^s^–1^)
340	2.30	0.92
370	6.13	3.36
390	9.24	6.35
***E***_***a***_**(kJmol**^**–1**^**)**	31.06	42.88

The MSD plots are in agreement with the Einstein relation, showing linearity over the time of the production run. The diffusion coefficients obtained using OPLS2005 are higher by a factor of 1.5–2.5 than OPLS3 depending on temperature, so mobility is significantly increased when the molecular interactions are represented with the OPLS2005 force field. From 340 to 390 K, an increase in the diffusivity by a factor of 4.0 and 6.9 was seen in the OPLS2005 and OPLS3 systems, respectively. The increase in diffusivity with temperature is greater in magnitude compared to the experiment where the rate of translational diffusion increased by only a factor of 2.8. The values of *D*_*s*_ are lower than those obtained by our QENS experiments, by an average factor of 3.5 and 7.0 for the OPLS2005 and OPLS3 simulations, respectively.

The activation energy was calculated from the Arrhenius plot in Figure S7. In both of the simulated systems, the activation energy was greater than the value obtained from QENS experiments by a factor of 1.4 and 1.9, using the OPLS2005 and OPLS3 models, respectively.

We note that the MSD plots from the MD simulations are calculated over 4 ns and thus probe a significantly different time scale to that covered by the IRIS spectrometer (ca. 100 ps).^52^ It is therefore not so surprising that the diffusion coefficients calculated from the total bulk translational motion over this much longer time scale do not agree exactly with those obtained over the shorter experimental time scale where more subtle jump diffusive motions may be observed in more detail. The MSD also takes into account all proton motions taking place in the 4 ns simulation, where our fitting of the QENS spectra was adequate with two Lorentzian functions approximating 2 average forms of motion over the time scale observed. A more appropriate comparison between experiment and our MD simulations takes place in the next section where we generate QENS observables upon sampling the MD simulation trajectories over the same time scale as the experiment and analyze them using the same method.

#### Incoherent Dynamic Structure Factor

**Figure 6:**
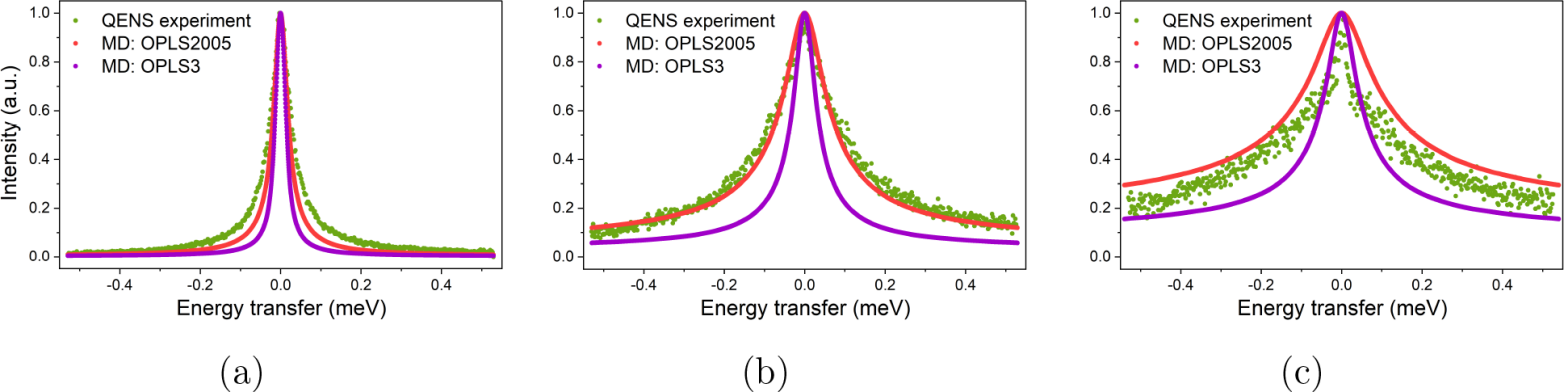
*S*_*inc*_(*Q*, ω)) from QENS experiments and MD simulations applying OPLS2005 and OPLS3 models for *p*-cresol at 370 K at (a) *Q* = 0.6, (b) *Q* = 1.2, and (c) *Q* = 1.6 Å^–1^.

To provide a direct comparison between the QENS experiment and the MD simulations, the fitted QENS spectra (*S*_*inc*_(*Q*, ω)) were compared to the *S*_*inc*_(*Q*, ω) calculated for each MD system at approximately equivalent *Q* values, as outlined in the Methods section. Each *S*_*inc*_(*Q*, ω) was then fit by Lorentzian functions to characterize and quantify the modes and rates of motion occurring in the simulations. To qualitatively assess the agreement between the simulated and experimental incoherent dynamic structure factors, they have been normalized and overlaid at various values of *Q*, as shown in Figure 6.

This direct comparison of the normalized structure factors indicates a better agreement between the OPLS2005 MD model and the experiment, with more broadening seen than the OPLS3 model. However, as mentioned in the experimental analysis, the QENS spectra required the fitting of a delta function due to the contribution of the aluminum can to the signal, which is not required by the spectra generated from MD simulation. Therefore, the fittings of the Lorentzian functions used to fit the MD generated *S*_*inc*_(*Q*, ω) are more appropriate to quantitatively compare and analyze the modes and rates of each type of motion occurring within the MD simulations.

**Figure 7:**
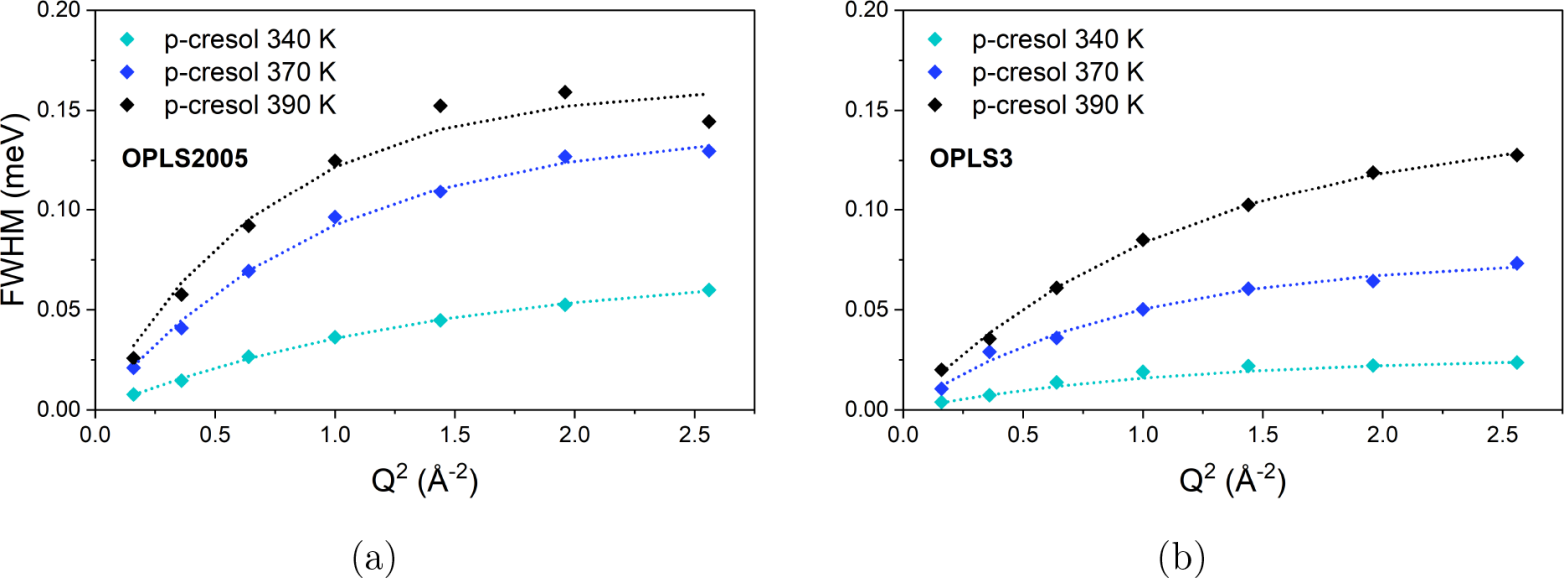
*Q* dependence of the fwhm of Lorentzian 1 fit to the *S*_*inc*_(*Q*, ω) calculated from MD simulations of *p*-cresol from 340 to 390 K applying (a) OPLS2005 and (b) OPLS3 models. The *Q* dependence is fit by the model of HR jump diffusion.

As with the experimental QENS spectra, two Lorentzians and a flat background were required to fit the *S*_*inc*_(*Q*, ω) of each MD model. The fittings for two values of *Q* for both MD models are shown in the Supporting Information in Figures S8 and S9. The same analysis was performed as with the QENS experiment, whereby the fwhm of each of the fitted Lorentzians was plotted against *Q*^2^, as shown in Figures 7 and 9.

**Figure 8:**
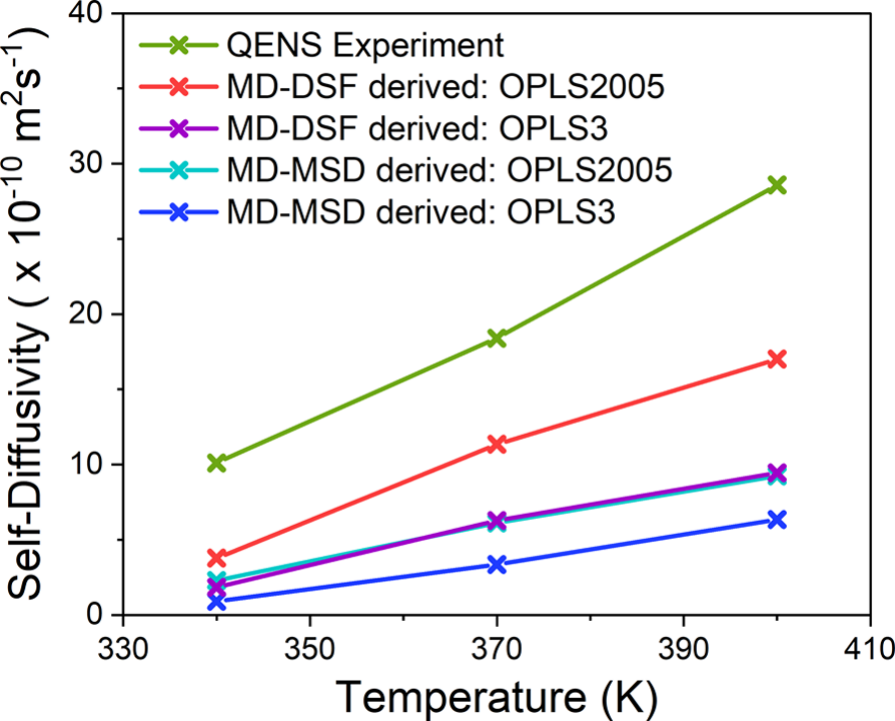
Jump diffusion coefficients from QENS experiments and calculated from simulations applying OPLS2005 and OPLS3 models for *p*-cresol from 340 to 390 K.

The diffusion coefficients obtained from the fwhm plots in Figure 7 are listed in Table 1 (MD-DSF derived *D*_*s*_) and are slower by an average factor of 2.0 for OPLS2005 and 3.8 for OPLS3 systems, illustrated by a comparison of the *D*_*s*_ values with temperature shown in Figure 8. For almost every temperature, the average residence time was greater in the simulations compared to the experiment. The residence times for the OPLS2005 systems provided a much closer match to the experiment being larger by only a factor of 1.1 compared to 2.1 for the OPLS3 systems, likely due to stronger intermolecular forces. In both simulated systems, the jump distances were similar to one another but shorter than the jumps within the experiment by factors of 1.3 and 1.4 for the OPLS2005 and OPLS3 systems, respectively, which may be compounded by increased system densities.

An overestimation of the intermolecular forces present in the simulations compared to experiment would cause this observed reduction in the diffusion rates, longer periods of residency, and shorter jump distances. There appears to be stronger intermolecular forces present in the OPLS3 systems compared to the OPLS2005 simulations (probed directly later in the paper) causing a reduction in the rates of diffusion, as seen in the MSD plots in Figure 5. Due to the greater oxygen to hydrogen charge difference of the molecules in the OPLS3 systems, these stronger forces are likely present in the form of stronger hydrogen bonding. The higher density of the OPLS3 systems (Figure S2) likely also plays a role.

The activation energies for jump diffusion, shown in Table 1, are also more similar to those of the experiment in comparison to those of the MSD calculations. For the OPLS2005 and OPLS3 models, the *E*_*a*_ values are larger by a factor of 1.5 and 1.6, respectively, calculated using the Arrhenius plot in Figure S9a. The increase in the activation energies for diffusion is commensurate with the aforementioned lower rates of diffusion, with more energy required to initiate translation, likely due to stronger intermolecular forces than those present in the experiment. As with the MSD results, the value of *E*_*a*_ for jump diffusion was larger for the OPLS3 simulations.

**Figure 9:**
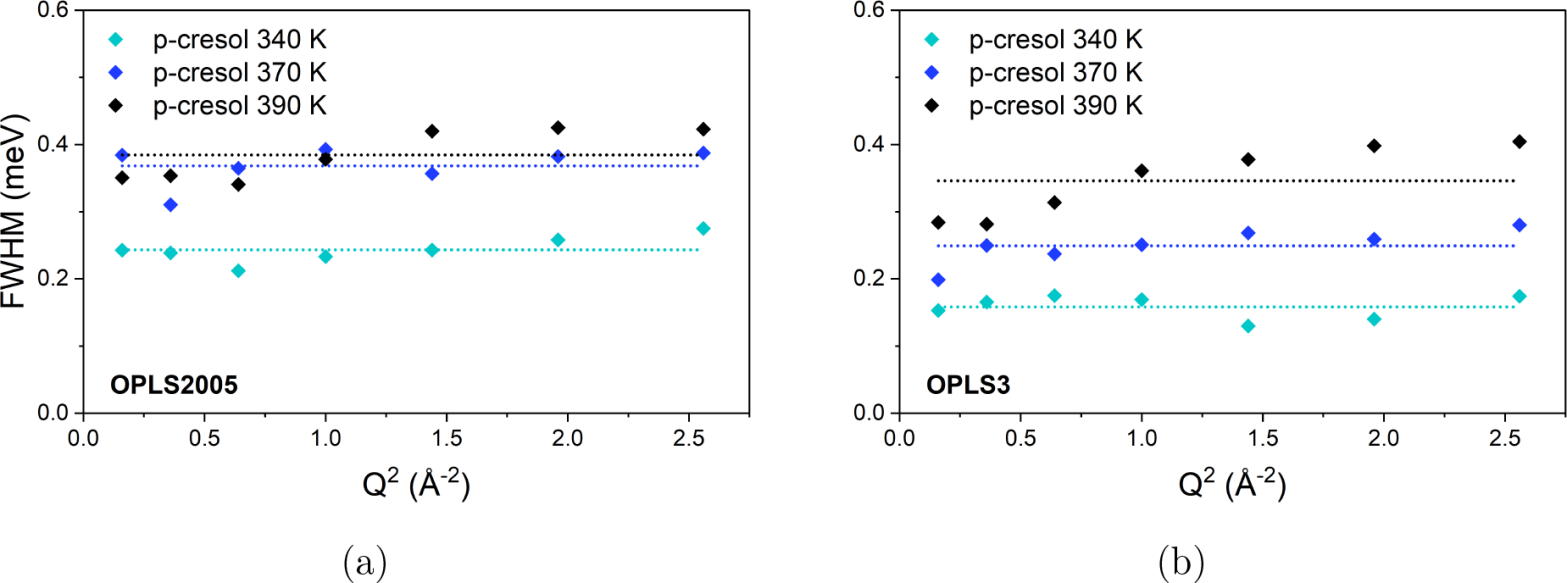
*Q* dependence of the fwhm of Lorentzian 2 fit to the *S*_*inc*_(*Q*, ω) calculated from MD simulations of *p*-cresol from 340 to 390 K applying (a) OPLS2005 and (b) OPLS3 models.

The fwhm of Lorentzian 2 fitted to the simulated *S*_*inc*_(*Q*, ω) shows no trend with *Q*^2^ similar to the second broader Lorentzian fit to the QENS spectra shown in Figure 3. As with the experimental data, the average width was used to calculate the isotropic rotational diffusion coefficient. The rotational rates are listed in Table 2.

The rates of rotation are lower in both of the simulations by an average factor of 1.2 and 1.6 for the OPLS2005 and OPLS3 simulations, respectively, and the activation energies for rotation (calculated from the Arrhenius plot in Figure S9b) are only marginally larger when applying the OPLS2005 model, but larger by a factor of 1.7 with the OPLS3 model. We again consider that the rotation of the “linear” *p*-cresol molecule is likely hindered by stronger intermolecular forces compared to the experiment and possibly is sterically hindered by surrounding cresol molecules due to higher system densities. The results align with the translational rates, where the use of the OPLS2005 model leads to a higher observed mobility than that of the OPLS3 model. Again, the slower rates and higher activation energy of the simulations applying the OPLS3 model compared to OPLS2005 can be explained by stronger polar cohesive interactions.

While the trends between the different simulated systems remain the same when comparing the MD-MSD and MD-DSF derived values of *D*_*s*_, the differences in the magnitude of the bulk properties are likely due the differences in the time scales probed and the errors associated with either method. Probing time scales of 4000 ps for the MSD analysis will give a more accurate analysis of the bulk translation, compared to the incoherent DSF analysis where only ca. 100 ps time scales were probed to match the resolution of IRIS. The method applied to model the DSFs has associated approximations, and the fitting of two Lorentzians cannot take into account every single mode of motion that may be occurring (as taken into account when calculating the MSD). There are also uncertainties involved with the relative weighting of each Lorentzian associated with either rotational or translational dynamics. Therefore, a combination of these two analyses must be considered when comparing classical simulations to the QENS experiments at this resolution.

### Hydrogen Bonding

**Figure 10:**
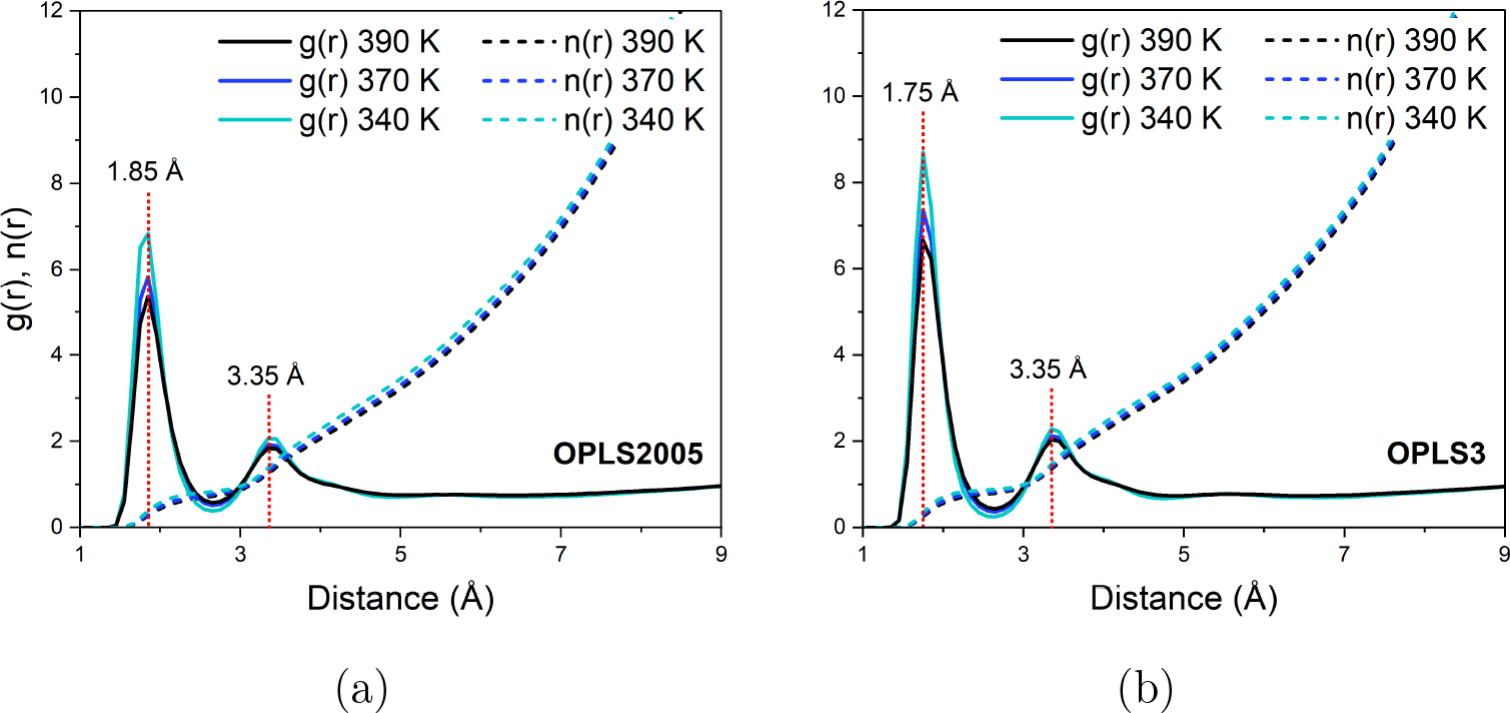
RDFs (*g*(*r*)) and coordination numbers (*n*(*r*)) of the *p*-cresol hydroxyl oxygen and hydrogen on different molecules from 340–390 K applying (a) OPLS2005 and (b) OPLS3 models.

The interactions between the hydroxyl oxygen and hydrogen atoms on different cresol molecules representing hydrogen bonding contribute to the cohesive energy observed to limit diffusion. The RDF plots between oxygen and hydrogen atoms of the cresols in each modeled system (Figure 10) illustrate differences in the H–O interactions at different temperatures and between the two models.

**Figure 11:**
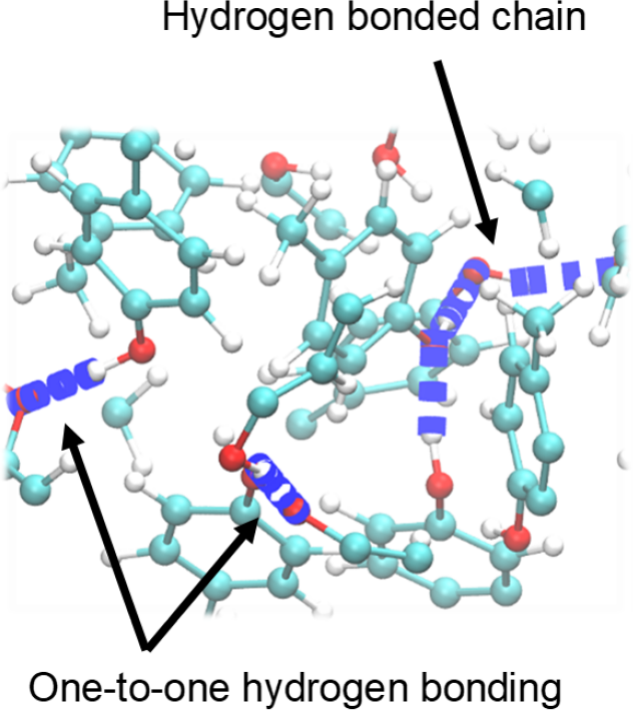
A snapshot of bulk *p*-cresol modeled by OPLS3 force fields at 340 K within a radius of ca. 6 Å and the hydrogen bonding networks recognized by VMD software.

**Figure 12:**
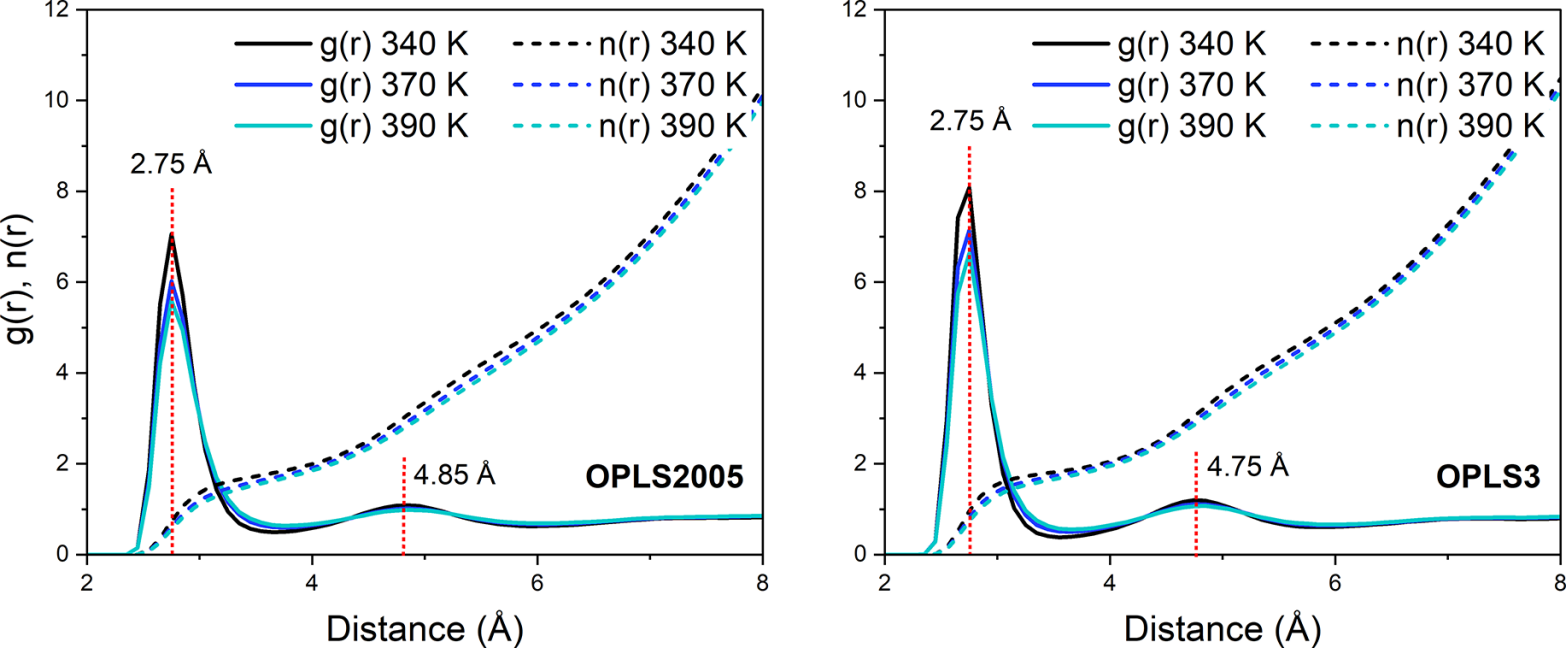
RDFs (*g*(*r*)) and the associated running integration numbers (*n*(*r*)) of the *p*-cresol hydroxyl oxygen on different molecules from 340 to 390 K applying (a) OPLS2005 and (b) OPLS3 models.

For both systems, significant interaction was observed from 1.75 to 1.85 Å, similar to that of liquid water,^53,54^ followed by a peak at ca. 3.4 Å, both labeled in Figure 10. The more intense peak at very close distances suggests that cresols frequently form a hydrogen bonded conformation within the simulated liquid which becomes more apparent as the temperature decreases. From the integral (*n*(*r*)), we can calculate the approximate coordination number associated with each peak. The first peak in the RDF plot accounts for approximately a one-to-one coordination, with an average *n*(*r*) = 0.85 for the OPLS3 systems and slightly less for the OPLS2005 systems at *n*(*r*) = 0.79. The second peak accounts for coordination to two adjacent molecules (*n*(*r*) = 2.0 for both systems). Different types of hydrogen bonding interactions can be observed in Figure 11, where a single frame of a section of the OPLS3 system at 340 K is shown. In both simulations, the coordination number shows a slight decrease with the temperature as the liquid becomes less ordered. Figure 10 highlights a more intense peak at shorter O–H bonding distances in the OPLS3 systems at 1.75 Å as opposed to the peak at 1.85 Å in the OPLS2005 systems. This corroborates with there being stronger and slightly shorter hydrogen bonding interactions, and therefore also a greater quantity of interactions in the OPLS3 systems, leading to the slower average translational and rotational rates observed, and higher energies of activation. The same trends were observed with the O–O distances on different cresol molecules, shown in Figure 12.

The peaks are broader compared to those in Figure 10. The first peak corresponds to average *n*(*r*) values of 1.60 and 1.71 for the OPLS2005 and OPLS3 systems, respectively, which is approximately 2 times that of the initial H–O shell. When investigating the number of specific hydrogen bonding interactions, a hydrogen bond was defined by the criteria of an O–O distance of <3.5 Å and with linear bonding angles of 180° ± 20. For each of the simulated systems, the average number of hydrogen bonds per molecule per time step is listed in Table 4.

**Table 4:** Mean Number of Hydrogen Bonds Formed per *p*-Cresol Molecule from 340–390 K Applying OPLS2005 and OPLS3 Models

	Mean number of hydrogen bonds per molecule	
Temperature (K)	OPLS2005	OPLS3
340	0.76	0.89
370	0.63	0.74
390	0.57	0.67

Over the temperature range, the average number of hydrogen bonds per molecule decreased by a factor of 1.3 using both force fields, as expected due to the increased kinetic energy of each molecule. There is a larger oxygen to hydrogen atom charge difference in the OPLS3 model (illustrated in Figure S1c) compared to the OPLS2005 model (Figure S1b). In the OPLS3 systems, this likely leads to a larger and stronger hydrogen bonded network, and hence a higher number of O–H–O bonding interactions occur (1.2 times more hydrogen bonds form compared to the OPLS2005 systems), shown in Table 4. Furthermore, this likely accounts for some of the observable differences seen between the different systems in the RDF analysis and the calculated values of *D*_*s*_ and *D*_*r*_.

**Figure 13:**
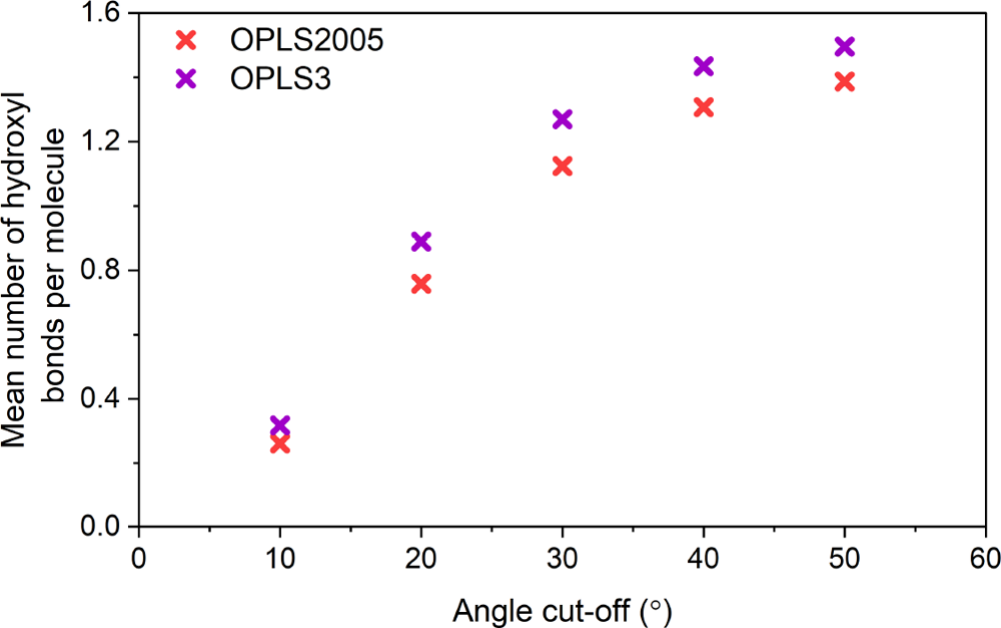
Mean number of hydrogen bonds per molecule with an increasing angular cutoff of 10–60° away from a linear O–H–O angle for the OPLS2005 and OPLS3 systems at 340 K.

The average O–H–O coordination numbers (*n*(*r*)) are approximately 2.5 and 2.3 times larger than the mean number of hydrogen bonds per time step for the OPLS2005 and OPLS3 systems, respectively, averaged across all temperatures. The difference between the value of *n*(*r*) and the mean number of hydrogen bonds also increases with temperature by a factor of ca. 1.7 from 340 to 390 K. The value of *n*(*r*) is expected to be larger than the number of hydrogen bonds because it accounts for molecules of all orientations and does not have the additional constraint of being within 180° ± 20 and so can account for various other conformations, such as parallel and offset parallel stacking. This can be rationalized through an increase in the number of hydrogen bonds observed with an increase in the bonding angle criteria demonstrated in Figure 13. The stronger O–H interactions in the OPLS3 systems are evident with more hydrogen bonding occurring as a proportion of the total molecules within coordinating distance compared to the OPLS2005 systems.

Figure 13 shows the importance of the interactions between atoms not in a hydrogen bonded conformation as well as the relative density of the liquid, while also highlighting the importance of the O–H interactions. Approximately 50% of the first coordination shell of molecules fall within a bonding angle of 180° ± 20. Bonding present at less stringent angular criteria is likely a result of nonideal tessellation of molecular bonding compared to hydrogen bonded water molecule networks.

## Conclusions

The dynamical behavior of liquid *p*-cresol was studied using QENS combined with MD simulations to understand the behavior of an important model component of biomass pyrolysis oil, where the accuracy of two force field models, OPLS2005 and OPLS3, was assessed through direct comparison with dynamical observables from QENS experiments.

The QENS experiments observed jump diffusion with coefficients from 10.1 to 28.6 × 10^–10^ m^2^s^–1^ and local isotropic rotational rates from 5.7 to 9.2 × 10^10^ s^–1^, with activation energies of 22.7 kJmol^–1^ and 10.1 kJmol^–1^ respectively.

MD simulations employing both models gave slower long-range diffusion rates than the experiment, with *D*_*s*_ values calculated from MSD plots from 2.3 to 9.2 × 10^–10^ m^2^s^–1^ for the OPLS2005 model and 0.9 to 6.4 × 10^–10^ m^2^s^–1^ for the OPLS3 model, which are lower than the experiment by factors of 3.5 and 7.0, respectively.

The simulated incoherent dynamic structure factors were then calculated, limited to the time scale of the QENS spectrometer to provide a direct comparison of the modes and rates of long-range and localized dynamics occurring in the MD simulations with the QENS experiment. From the *S*_*inc*_(*Q*, ω) fittings, the same dynamics observed in experiment (jump diffusion and isotropic rotation) were observed within both simulation models. The calculated jump diffusion coefficients were slower than the experiment from 3.8 to 17.0 × 10^–10^ m^2^s^–1^ for the OPLS2005 model and 1.9 to 9.4 × 10^–10^ m^2^s^–1^ for the OPLS3 model over the temperature range (different by a factor of ca. 2.0 and 3.8, respectively). Slower rates of rotation were also observed in the simulations by a factor of 1.2 and 1.6 respectively. We consider that the differences in rates are due to stronger intermolecular forces and higher system densities in the models, which are even more pronounced in the OPLS3 model with particularly strong “hydrogen bonding” observed.

The OPLS2005 model gives better agreement with the experimental diffusion and rotational rates. The differences in the rates between the two MD models may be largely attributed to the hydrogen to oxygen charge difference, where the greater charge difference in the OPLS3 model leads to a greater strength and frequency of “hydrogen bonding” and a slower rate of translational and rotational diffusion.

The study highlights the importance of assessing the accuracy of established models in probing even relatively simple liquid systems by comparison with experiments and measuring dynamics over the same time scales. This will be particularly important when probing components in far more complex mixtures, such as biomass pyrolysis oil. It also reveals how subtle changes in modeled molecular characteristics can lead to some notable consistencies and contrasts between types of motion and their modeled rates when compared to suitable experiments.

## Supporting Information Available

Potential parameters used for modeling the MD simulations, a comparison of simulated and experimental densities, an explanation of the models applied to fit the QENS and MD obtained dynamic structure factors and the Arrhenius plots required for calculating the activation energies (PDF)
