# Supplementary material for: Experimental and Modeling Studies of Local and Nanoscale para-Cresol Behavior: A Comparison of Classical Force Fields
Source: J Phys Chem A. 2023 Apr 11;127(15):3305–16. doi: 10.1021/acs.jpca.2c08022 (PMC10123653; doi:10.1021/acs.jpca.2c08022)
Supplement: Supplementary file 1 — jp2c08022_si_001.pdf [file jp2c08022_si_001.pdf]

Supporting Information
Experimental and Modelling Studies of Local and Nanoscale *para*-cresol Behaviour: a Comparison of Classical Forcefields.

Katie S. C. Morton,[†,‡] Alin M. Elena,[¶] Jeff Armstrong,[*,§] and Alex J. O'Malley[*,†,‡]

†*Centre for Sustainable and Circular Technologies, Department of Chemistry, University of Bath, UK, BA2 7AY*

‡*UK Catalysis Hub, Research Complex at Harwell, Science and Technology Facilities Council, Rutherford Appleton Laboratory, Oxford, OX11 0FA, UK*

¶*Daresbury Laboratory, STFC, Daresbury, WA4 4AD, UK*

§*ISIS Pulsed Neutron and Muon Facility, Science and Technology Facilities Council, Rutherford Appleton Laboratory, Didcot, OX11 0QX, UK*

E-mail: jeff.armstrong@stfc.ac.uk; a.o'malley@bath.ac.uk

Setting up the molecular dynamics simulations

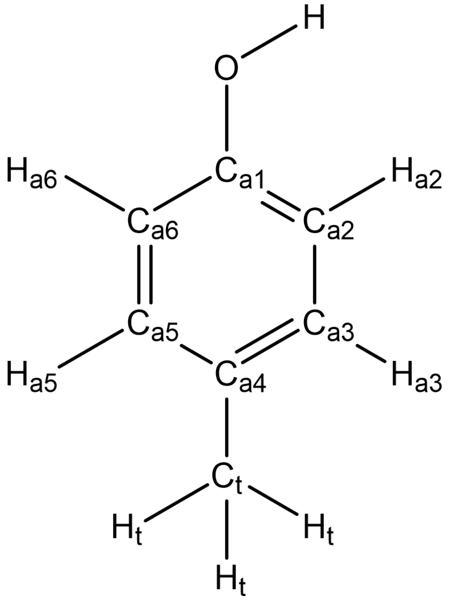

(a)

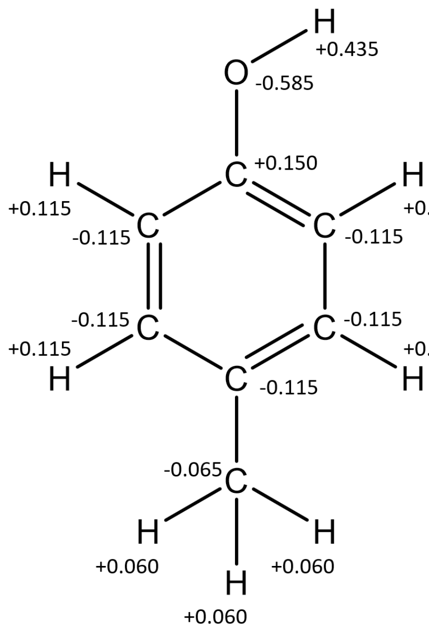

(b)

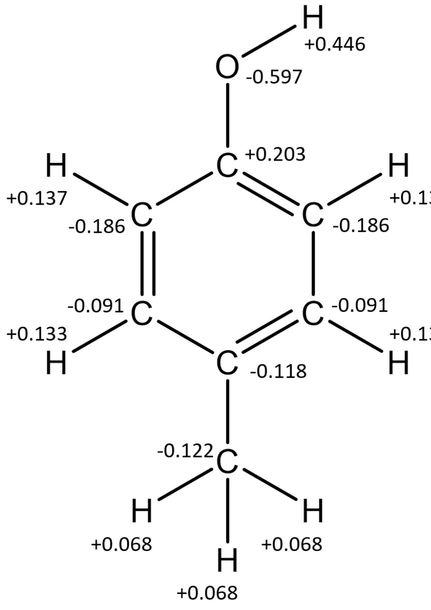

(c)

Figure S1: A molecule of *p*-cresol with the atoms labelled with (a) the atom names according to table S1, (b) the atom charges of the OPLS2005 model and (c) the atom charges of the OPLS3 model.

Table S1: Potential parameters describing *p*-cresol intra- and intermolecular forces.
*SF are the 1-4 interaction scale factors.

**Harmonic bond**, $U(r_{ij}) = \frac{k}{2}(r_{ij} - r_0)^2$		
Bond	k (kcalmol^{-1} Å^{-2})	r_0 (Å)
H-O	1106	0.945
O-C_a	900	1.364
C_a-C_a	938	1.400
C_a-H_a	734	1.080
C_a-C_t	634	1.510
C_t-H_t	680	1.090

**Harmonic bind angle**, $U(\theta_{ijk}) = \frac{k}{2}(\theta_{ijk} - \theta_0)^2$		
Angle	k (kcalmol^{-1} rad^{-2})	θ_0 (°)
H-O-C_a	70	113
O/C_t-C_a-C_a	140	120
C_a-C_a-C_a	126	120
C_a-C_a-H_a	70	120
C_a-C_t-H_t	70	109.5
H_t-C_t-H_t	66	107.8

**Triple cosine dihedral**, $U(\phi_{ijkl}) = \frac{1}{2}[A_1(1 + \cos(\phi_{ijkl})) + A_2(1 - \cos(2\phi_{ijkl})) + A_3(1 + \cos(3\phi_{ijkl}))$					
Dihedral	A_1 (kcalmol^{-1})	A_2 (kcalmol^{-1})	A_3 (kcalmol^{-1})	Electrostatic SF*	Van der Waals SF*
H-O-C_a-C_a	0.0	1.682	0.0	0.5	0.5
H_a/C_a-C_a-C_a-C_a/H_a/C_t/O	0.0	7.250	0.0	0.0	0.0
C_a-C_a-C_t-H_t	0.0	0.000	0.0	0.5	0.5
C_t-C_{a4}-C_{a3}-C_{a5}, O-C_{a6}-C_{a1}-C_{a2}	0.0	8.000	0.0	0.0	0.0
C_{a4}-C_{a6}-C_{a5}-H_{a5}, C_{a4}-C_{a2}-C_{a3}-H_{a3}, C_{a5}-C_{a1}-C_{a6}-H_{a6}, C_{a3}-C_{a1}-C_{a2}-H_{a2}	0.0	2.200	0.0	0.0	0.0

**Lennard-Jones Potential**, $U(r_{ij}) = 4\epsilon_{ij}[\frac{\theta_{ij}}{r_{ij}}^{12} - \frac{\theta_{ij}}{r_{ij}}^{6}]$, $\epsilon_{ij} = (\epsilon_i \times \epsilon_j)^{\frac{1}{2}}, \theta_{ij} = \frac{1}{2}(\theta_i + \theta_j)$				
Atom	OPLS2005: q (e^-)	OPLS3: q (e^-)	ϵ_i ($kcalmol^{-1}$)	θ_i (Å)
H	0.435	0.4456	0.030	0.50
O	-0.585	-0.5970	0.170	3.07
C_{a1}	0.150	0.2025	0.070	3.55
C_{a2}, C_{a6}	-0.115	-0.1863	0.070	3.55
C_{a3}, C_{a5}	-0.115	-0.0908	0.070	3.55
C_{a4}	-0.115	-0.1179	0.070	3.55
C_t	-0.065	-0.1220	0.066	3.50
H_{a2}, H_{a6}	0.115	0.1368	0.030	2.42
H_{a3}, H_{a5}	0.115	0.1333	0.030	2.42
H_t	0.060	0.0676	0.030	2.50

The experimental densities were measured on a DMA 4100 M density meter and showed close agreement with values found in the literature.[1–3] The experimental densities were only measured up to 363 K due to limitations of the instrument. These densities were compared to the densities of both simulated systems. The MD system densities were obtained from the average density after running each system for 2 ns in an NPT ensemble following an equilibration procedure. All the density values are listed in table S2. A plot of the variation in densities with temperature is displayed in figure S2.

Table S2: The densities of liquid *p*-cresol.

	**Density** (gcm^{-3})			
**Temperature** (K)	**Literature**	**Experimental**	**OPLS2005**	**OPLS3**
260			1.061	1.069
293		1.034		
298	1.030			
303		1.026		
310			1.027	1.042
313	1.019	1.019		
323	1.011	1.011		
333	1.004	1.003		
340		0.997	0.999	1.020
343	0.996	0.994		
353	0.988	0.986		
363	0.980	0.978		
370			0.970	0.993
390			0.951	0.975

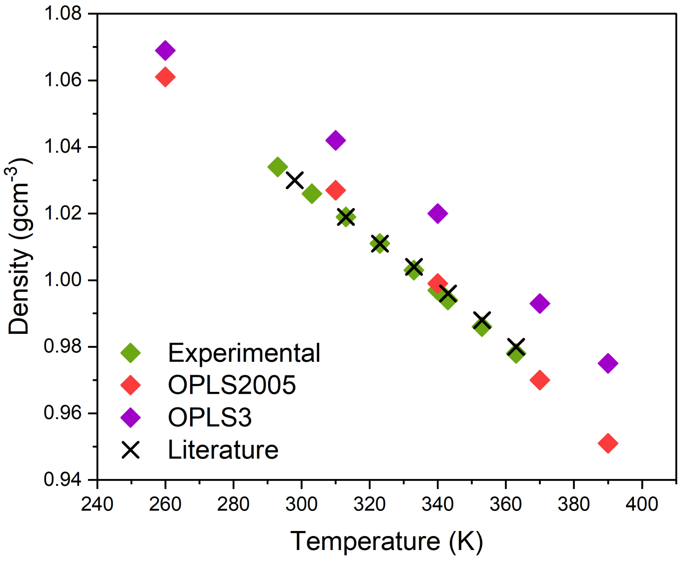

Figure S2: The densities of liquid *p*-cresol.

The OPLS2005 system densities give a close match to the experimental densities, especially at higher temperatures. The greater density of the OPLS3 systems highlights potential inaccuracies with the intramolecular force-fields and/or the cresol atomic charges.

Quasielastic neutron scattering

Significant broadening of the QENS spectra is shown for *p*-cresol at 370 K across four values of Q in figure S3.

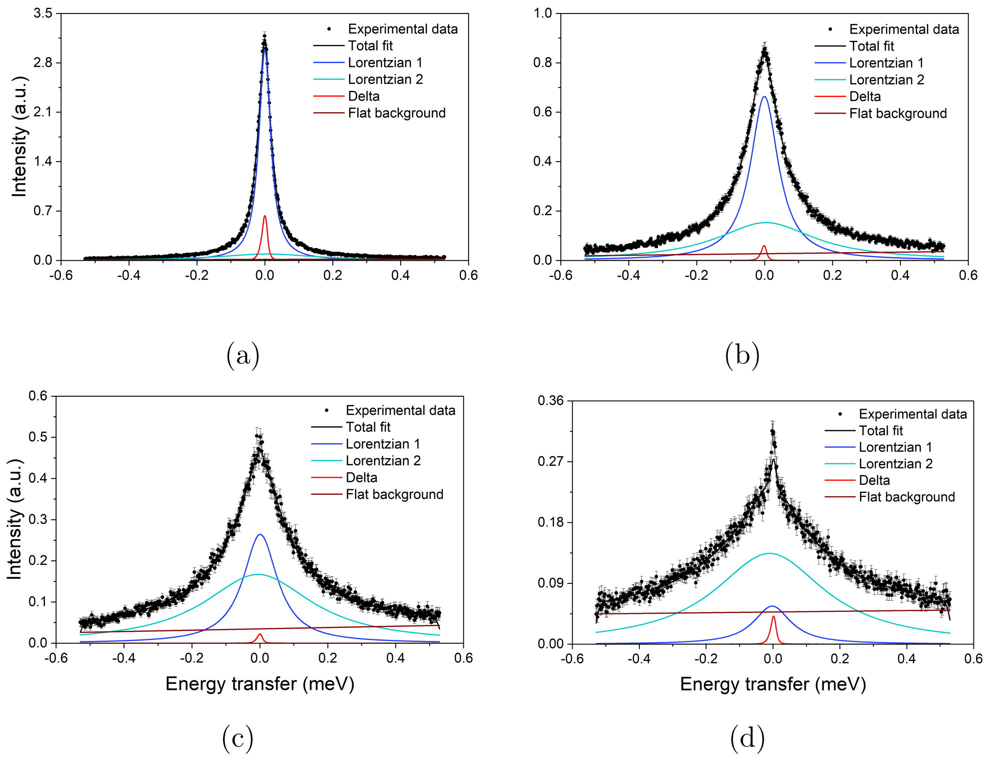

Figure S3: QENS spectra for *p*-cresol at 370 K at (a) $Q = 0.48$, (b) $Q = 0.85$, (c) $Q = 1.17$ and (d) $Q = 1.53$ Å^{-1}. Two Lorentzian functions were required to give a good fit to the quasielastic broadening.

Modelling localised and translational motions

For modelling localised motions, only confined motions and isotropic rotations of the molecules were considered in depth. Uniaxial rotations were considered unlikely, as the molecules are likely to rotate in multiple orientations in a liquid and methyl rotations were considered to rotate on a timescale that was too fast for the instrument to measure, considering previous data,[4] and may be partially accounted for by the flat background.

A cresol molecule translating within a confined space such as a dynamical basin can be described by a confined diffusion model, depicted in S4 (a). Confined translation assumes that the potential field within a symmetrical sphere is low compared to the infinite potential outside of it. Confined diffusion is indicated by a fixed Lorentzian half-width half-maxima (HWHM,$\Delta\omega(Q)$) at low Q^2, corresponding to a lack of movement over long distances, but follows Fickian diffusion at higher Q^2. The diffusion coefficient (D_s) for confined diffusion

can then be calculated. In our results, the Lorentzian full-width half-maxima (FWHM = HWHM × 2) was plot against Q^2.

$$\Delta\omega(Q) = \frac{4.33 D_s}{{r_{conf}}^2} \tag{1}$$

Isotropic rotation, describes a molecule's random re-orientation around any axis, shown in figure S4 (b). The HWHM of the Lorentzians corresponding to localised motions do not follow a linear trend with Q^2. The rate of isotropic rotation is calculated from the average of the HWHM values.

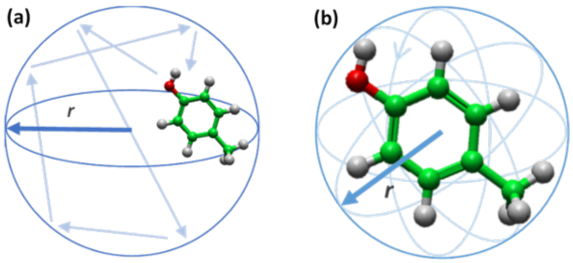

Figure S4: Various motions of a *p*-cresol molecule: (a) confined translational motion and (b) isotropic rotation

.

For purely diffusive motions, Fickian diffusion shows linear proportionality between the Lorentzian broadening and Q^2, whereas jump diffusion begins to reach a plateau at higher Q^2. During jump diffusion the molecule translates a certain distance (d) between time periods (τ) where the molecule appears static and it is vibrating around an equilibrium position. Jump diffusion often occurs in liquid systems with local order where the translation of molecules is periodically hindered by sterics or intermolecular interactions. Fickian diffusion is modelled below.

$$\Delta\omega(Q) = D_s Q^2 \tag{2}$$

Various jump diffusion models can be used to fit suitable Lorentzian HWHM plots. The Hall and Ross (HR)[5] and Singwi and Sjölander (SS)[6] models represent jump lengths as a

continuous distribution developed for the characterisation of liquids. This is in opposition to the Chudley and Elliot (CE)[7] model which assumes discrete jump diffusion distances which was deemed unsuitable for largely disordered liquid diffusion, and so it is not discussed here. All of the models assume that the jump time is negligible.

The HR model is as follows.

$$\Delta\omega(Q) = \frac{1}{\tau}(1 - \exp{(-\frac{Q^2\langle d^2\rangle}{6}))} \tag{3}$$

$$D_s = \frac{d^2}{2\tau} \tag{4}$$

The jump lengths are distributed normally according to the equation below.

$$\rho(d) = \frac{2d^2}{d_0^3(2\pi^{\frac{1}{2}}} \exp(-\frac{d^2}{2d_0^2}) \tag{5}$$

Whereas, the SS model is as follows.

$$\Delta\omega(Q) = \frac{1}{6\tau}(\frac{Q^2\langle d^2\rangle}{1 + \frac{Q^2\langle d^2\rangle}{6}}) \tag{6}$$

$$D_s = \frac{d^2}{\tau} \tag{7}$$

Here, the jump lengths are characterised by an exponentially decaying distribution.

$$\rho(d) = \frac{d}{d_0^2} \exp(-\frac{d}{d_0}) \tag{8}$$

Experimental QENS analysis

The FWHM of Lorentzian 1 relating to translational diffusion exhibited linear proportionality at low Q^2 but reached a plateau as Q^2 increased, typical of a jump-diffusion process. Little difference was observed qualitatively in the goodness of fit for either the HR or SS model,

shown in figure S5. However, the HR model was chosen on the basis of it having higher mean coefficients of determination (R^2) and lower χ^2 values. The R^2 values were calculated as 0.987 and 0.930 and the χ^2 values as 0.114 and 0.189 for the HR and SS models respectively, averaged across all temperatures.

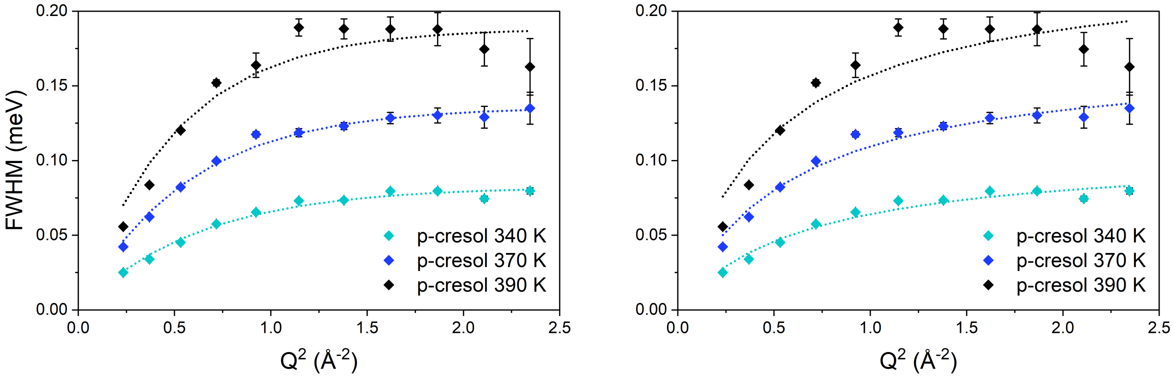

(a) (b)

Figure S5: Obtained from QENS spectra of *p*-cresol from 340–390 K, the Q-dependence of the FWHM of Lorentzian 1 fit by a model of (a) HR and (b) SS jump diffusion.

The diffusion coefficients for both types of dynamics observed in the QENS experiments were used to calculate their respective activation energies E_a via the Arrhenius plots shown below.

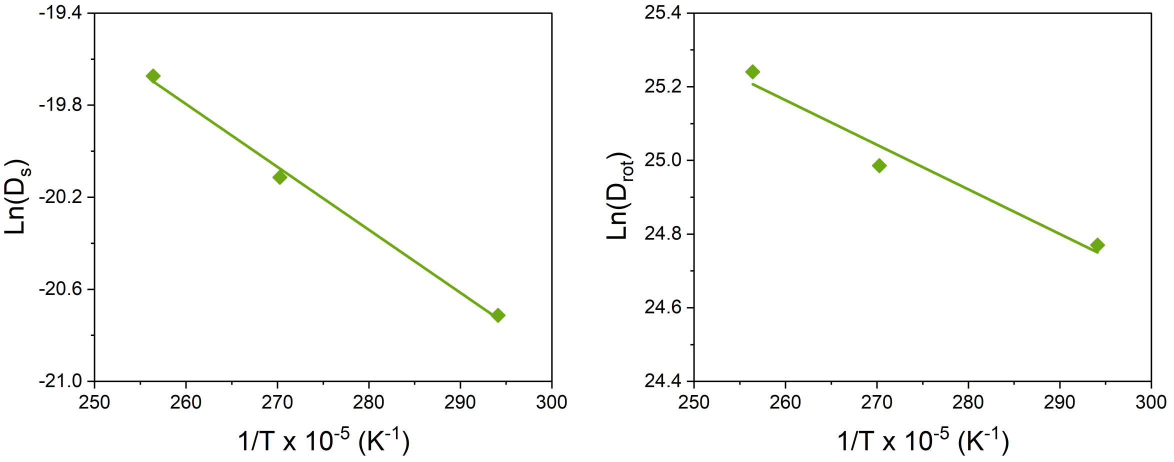

(a) (b)

Figure S6: Arrhenius plots used to calculate the E_a from the QENS experiments on *p*-cresol for (a) translational jump diffusion and (b) isotropic rotational diffusion.

Molecular dynamics simulations

Mean squared displacement

The D_s for translational diffusion obtained from the MSD plots for each system applying the two force-fields were used to calculate their respective activation energies E_a via the Arrhenius plot shown in figure S7.

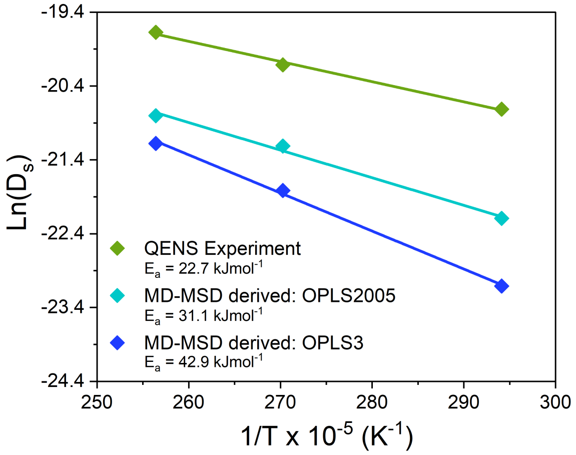

Figure S7: Arrhenius plot used to calculate the E_a of translation calculated from the MSDs of simulated *p*-cresol systems applying OPLS2005 and OPLS3 models.

Incoherent dynamic structure factor

For the full details of the background, theory and calculations applied to gain the incoherent dynamic structure factors from the simulated trajectories, we direct the reader to the MDANSE manual version 1.0, through the following link:

https://epubs.stfc.ac.uk/work/51935555 [8]

The calculated incoherent dynamic structure factors for the OPLS2005 and OPLS3 systems at 370 K were then fit using DAVE software,[9] with the fittings shown in figures S8 and S9.

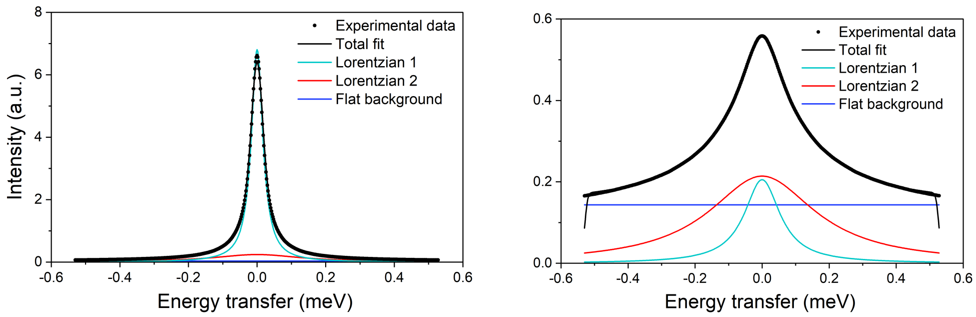

(a) (b)

Figure S8: Simulated $S_{inc}(Q,\omega)$ for p-cresol at 370 K applying the OPLS2005 model at (a) Q = 0.6 and (b) Q = 1.6 Å^{-1}. Two Lorentzian functions were required to give a good fit to the quasielastic broadening.

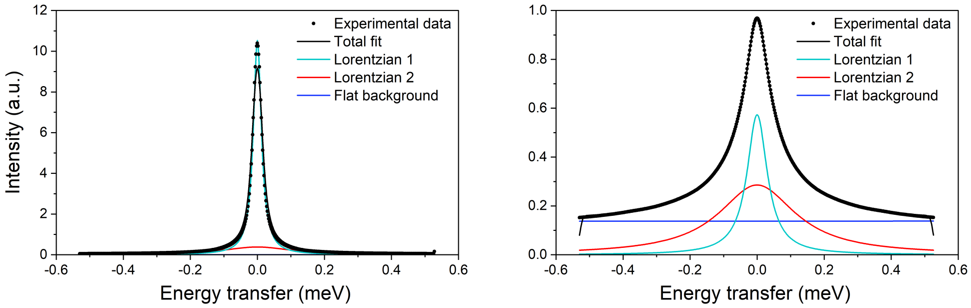

(a) (b)

Figure S9: Simulated $S_{inc}(Q,\omega)$ for p-cresol at 370 K applying the OPLS3 model at (a) Q = 0.6 and (b) Q = 1.6 Å^{-1}. Two Lorentzian functions were required to give a good fit to the quasielastic broadening.

The Arrhenius plots for translational jump diffusion and rotation obtained from the fittings to the simulated $S_{inc}(Q,\omega)$ for each system applying the two force-fields (alongside the values obtained from the QENS experiment for reference) were used to calculate their respective activation energies via the Arrhenius plots shown in figure S10 (a) and (b).

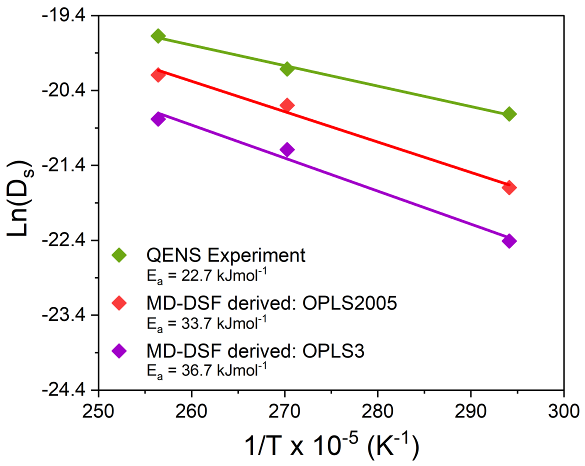

(a)

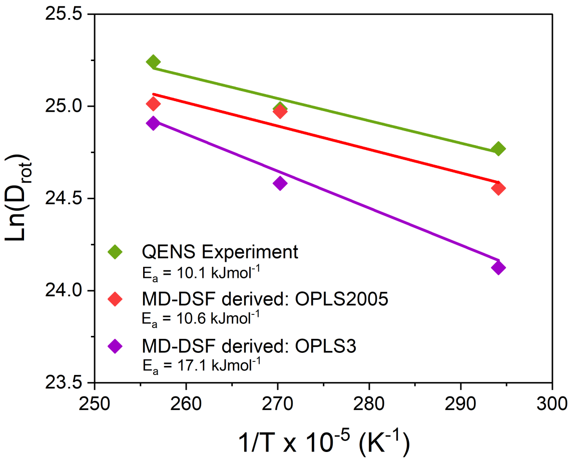

(b)

Figure S10: Arrhenius plots used to calculate the E_a from the incoherent dynamic structure factors obtained from QENS experiments and MD simulations applying OPLS2005 and OPLS3 models of *p*-cresol (a) translational jump diffusion and (b) isotropic rotation.

References

(1) Elizalde-Solis, O.; García-Fuentes, D.; Álvarez-Badillo, S.; Zúñiga-Moreno, A.; Camacho-Camacho, L. E.; Galicia-Luna, L. A. Densities of Cresols and Linear Alkane Mixtures at High Pressure. *Journal of Chemical and Engineering Data* **2013**, *58*, 2163–2175.

(2) Cunha, D. L.; Coutinho, J. A. P.; Daridon, J. L.; Reis, R. A.; Paredes, M. L. L. Experimental Densities and Speeds of Sound of Substituted Phenols and Their Modeling with the Prigogine–Flory–Patterson Model. *Journal of Chemical and Engineering Data* **2013**, *58*, 2925–2931.

(3) Bhatia, S. C.; Rani, R.; Bhatia, R. Densities, speeds of Sound, and Refractive Indices of Binary Mixtures of Decan-1-ol with Anisole, o-Cresol, m-Cresol, and p-Cresol at iT/i = (298.15, 303.15, and 308.15) K. *Journal of Chemical and Engineering Data* **2010**, *56*, 1669–1674.

(4) Hernandez-Tamargo, C.; O'Malley, A.; Silverwood, I. P.; de Leeuw, N. H. Molecular behaviour of phenol in zeolite Beta catalysts as a function of acid site presence: a quasielas-

tic neutron scattering and molecular dynamics simulation study. *Catalysis Science and Technology* **2019**, *9*, 6700–6713.

(5) Hall, P. L.; Ross, D. Incoherent neutron scattering functions for random jump diffusion in bounded and infinite media. *Molecular Physics* **1981**, *42*, 673–682.

(6) Jobic, H. On the jump diffusion of molecules in zeolites measured by quasi-elastic neutron scattering. *Microporous and Mesoporous Materials* **2002**, *55*, 159–169.

(7) Chudley, C. T.; Elliott, R. J. Neutron Scattering from a Liquid on a Jump Diffusion Model. *Proceedings of the Physical Society* **1961**, *77*, 353–361.

(8) Goret, G.; Aoun, B.; Pellegrini, E. MDANSE: An Interactive Analysis Environment for Molecular Dynamics Simulations. *Journal of Chemical Information and Modeling* **2017**, *57*, 1–5.

(9) Azuah, R. T.; Kneller, L. R.; Qiu, Y.; Tregenna-Piggott, P. L. W.; Brown, C. M.; Copley, J. R. D.; Dimeo, R. M. DAVE: A Comprehensive Software Suite for the Reduction, Visualization, and Analysis of Low Energy Neutron Spectroscopic Data. *Journal of Research of the National Institute of Standards and Technology* **2009**, *114*, 341.
